# A Preliminary Survey of Cultured Fusaria from Symptomatic Legume Grains in North-Eastern Poland

**DOI:** 10.3390/toxins11100569

**Published:** 2019-09-29

**Authors:** Maciej Żelechowski, Jacek Olszewski, Tomasz Kulik

**Affiliations:** 1 Department of Botany and Nature Protection, University of Warmia and Mazury in Olsztyn, Plac Łódzki 1, 10-727 Olsztyn, Poland; 2 Experimental Education Unit, Oczapowskiego 8, 10-719 Olsztyn, Poland; jacolsz@uwm.edu.pl

**Keywords:** *Fusarium*, qPCR, legume plants, common vetch, blue lupine, faba bean, white lupine

## Abstract

Legumes are amongst the most promising crops to satisfy the increasing demand for protein-rich food and feed. Today, however, their cultivation in Europe is low, while European agriculture faces a deficit of protein-rich feed, of which the largest part is met by imported soybean. It has been suggested that some legumes can at least partially substitute for soybean in different types of feed. Despite their benefits, legumes may also remain a significant concern to human and animal health, especially regarding grain contamination with Fusaria and their mycotoxins. In this study, we determined the species composition of *Fusarium* field isolates recovered from diseased grains of various legumes. Our results showed that *Fusarium avenaceum* was mainly responsible for grain deterioration of common vetch, faba bean, and blue lupine. Besides, we found that *F. equiseti* also appeared to be a major pathogen of common vetch. This study is the first ever to report common vetch as a host for F. *tricinctum, F. equiseti*, and *F. graminearum* sensu stricto. Our results indicate that the composition of toxigenic Fusaria associated with grains of legumes is different than that previously observed in cereal grains.

## 1. Introduction

Legumes are amongst the most promising crops to satisfy the increasing demand for protein-rich food and feed [1]. Nowadays, they are the second most important food source after grasses and are a relatively better source of lysine and total proteins than cereals [2]. Today, however, the cultivation of legumes in Europe is low, while European agriculture faces a deficit of protein-rich feed, of which the largest part is met by imported soybean and soybean meal. It has been suggested that some legumes can at least partially substitute for soybean in different types of feed [3]. Sound evidence supports the health benefits of increasing legume intake by humans. Consumption of legumes implicated lowering risks of many diseases like heart disease, high blood pressure, stroke, and type 2 diabetes [4].

Despite their benefits, legumes may also remain a significant concern to human and animal health, especially regarding grain contamination with fungal biomass and mycotoxins. Fungi of the genus *Fusarium* may pose a serious problem due to their widespread occurrence and cosmopolitan range. The most common mycotoxins produced by Fusaria are trichothecenes, enniatins, zearalenone, and fumonisins, exerting various negative effects on humans and animals [5].

We searched the biomedical literature through the PubMed database with no date restrictions for case reports and outbreaks using the search terms “*Fusarium*”, “mycotoxins”, “faba bean”, “common vetch”, “blue lupine”, and “white lupine”. We have chosen these crops because they are suggested as the most promising alternatives for soybean protein [3]. Google Scholar was also searched with similar terms. We found that the literature reporting the incidence of toxigenic Fusaria on these alternative legume crops is scarce (Table 1). Today, most surveys characterizing seed-borne Fusaria in Europe come from studies that report the incidence of these pathogens on small-grain cereals. Although a broad range of *Fusarium* species may be associated with grasses, multiple surveys conducted over the last decade have provided strong evidence on the predominance of *F. graminearum* sensu stricto in various European localities [6].

Limited data on the incidence of Fusaria on legumes prompted us to investigate the presence of *Fusarium* spp. in legume grains harvested in North-Eastern Poland in the 2017/2018 growing seasons. Our preliminary results showed that *F. avenaceum* was mainly responsible for grain deterioration of common vetch, faba bean, and blue lupine. Previous surveys from cereals indicated F. avenaceum to be the major source of enniatins in plant-derived food, posing significant risk to food and feed safety [5]. In this study, we also recovered a high number of isolates of *F. equiseti* from common vetch. F. equiseti belongs to the *Fusarium incarnatum*-*equiseti* species complex with the potential to produce diverse mycotoxin compounds, such as type A trichothecenes and zearalenone [5]. These results indicate that the composition of toxigenic Fusaria associated with legume grains is different from that previously observed in cereal grains. This study reports the incidence of *F. tricinctum, F. equiseti*, and *F. graminearum* sensu stricto infecting common vetch for the first time ever.

## 2. Results and Discussion

225 legume grains showing visual symptoms of the fungal disease were selected to obtain *Fusarium* isolates for analyses. Diseased grains were shriveled, discolored, and/or covered by fungal mycelia. After incubation and visual selection of fungal colonies on potato dextrose agar (PDA), we obtained forty-three *Fusarium*-like cultures that were further subjected to real-time polymerase chain reaction (PCR) analyses.

We used different species–specific assays to identify the isolates to the species level. Thirty-seven out of the 43 isolates gave positive results enabling their quick assignment (Table 2, Table S1). 

Our results showed that *F. avenaceum* was mainly responsible for grain deterioration of common vetch, faba bean, and blue lupine (49% of isolates) (Table 3)*. F. avenaceum* is a common plant pathogen infecting a variety of hosts worldwide. In cereals, it is often responsible for the crown rot and head blight that affects yield and quality of grain [28]. Most research works documenting the incidence of this species on legume grains are relatively old and come mainly from Polish surveys [9,12,13]. *F. avenaceum* contaminates grain with enniatins [28]; however, according to our knowledge, no data is available on the contamination of legume grains with this group of mycotoxins. The ability to produce enniatins by Fusaria is governed by *esyn1* gene encoding a multifunctional enzyme enniatin synthetase [29]. In this study, we showed that all examined isolates of *F. avenaceum* harbored *esyn1* gene, which indicates their ability to produce enniatins. This highlights the need for further chemical studies to confirm the contamination of legume grains with these cyclic hexadepsipeptides. Previous Polish studies showed that besides *F. avenaceum*, *F. culmorum* was a pathogen occasionally associated with faba bean grains [12,13,14], but our results do not reveal the incidence of *F. culmorum* on any of the examined hosts. Recent studies on cereals have shown that *F. culmorum* has been displaced by *F. graminearum* s.s. as the major agent of Fusarium head blight (FHB) of wheat in Europe [6]. This dramatic shift has also been revealed in Poland [30]. Thus, the results obtained in this study may indicate previously undocumented loss of *F. culmorum* on legumes, suggesting that the reduction of *F. culmorum* incidence in grain-associated Fusaria may also occur in other non-cereal crops.

In our study, besides *F. avenaceum*, *F. equiseti* also appeared to be a major pathogen of common vetch. F. equiseti is a cosmopolitan soil-borne fungus that has been detected in roots and plant tissues worldwide [31]. A recent analysis conducted using genealogical concordance phylogenetic species recognition (GCPSR) has revealed that *F. equiseti* belongs to the *Fusarium incarnatum*-*equiseti* species complex (FIESC), consisting of at least 33 phylogenetically distinct species, grouped into two major clades: *Equiseti* and *Incarnatum* [32]. FIESC members are increasingly associated with diseases of numerous plants including Fusarium root rot in soybean [33]. They have also been associated with human and animal health problems [34]. In addition, *F. equiseti* has been identified in soybean grains; however, reports documenting its incidence on other legumes are mainly limited to old surveys (Table 1). According to our knowledge, this is the first report showing the incidence of F. equiseti on common vetch. Among 18 isolates recovered from this crop, a single isolate was identified as F. graminearum s.s. This phylogenetic species has been recently recognized as the major FHB member of wheat in Poland [30]. *F. graminearum* s.s. has been found to contaminate soybean grains worldwide, but its incidence on other legume crops has been reported only for faba bean [13]. The emergence of *F. graminearum* s.s. in Europe has been linked to increased production of maize, which favors ascospore formation, which survives in crop residues and may be carried over long distances [30]. Soybean residues were also found to support high levels of sporulation by *F. graminearum* s.s. [35]. The identified incidence of *F. graminearum* s.s., albeit occasional, could promote further more comprehensive studies evaluating the risk of ascospore production by this pathogen on other hosts apart from soybean legume residues. Overall, our results indicate that the composition of toxigenic Fusaria associated with grains of legumes is different from that previously observed in cereals [6]. The revealed high incidence of both *F. avenaceum* and *F. equiseti* needs to be confirmed on a larger scale by incorporating more samples from a wide geographic area. Our further work will aim at molecular characterization of the recovered isolates of *F. equiseti* as these strains may comprise phylogenetically distinct species having the potential to produce diverse mycotoxin compounds [32]. Our further work will also include characterization of Fusaria from soybean samples, as this crop is expected to be increasingly cultivated in the EU [36].

## 3. Materials and Methods

### 3.1. Legume Grain Samples

**Figure 1:**
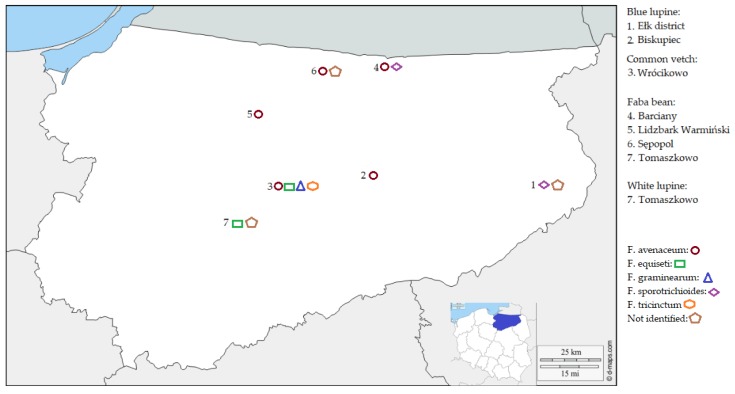
Locations of fields in Warmia-Mazury Province in Poland, from which legume grains were sampled for analyses

Grains with symptoms of fungal infection with purple/pink lesions and/or shriveled grains were selected from different 2017 and 2018 grain samples (0.5 kg) originating from seven different fields in the North-Eastern Poland (Figure 1). Diseased grains were placed in Petri dishes with distilled water and kept for 24 hours at room temperature. After soaking, grains were surface sterilized with 70% ethanol (EtOH) for 2 min and placed on PDA medium. Grains were incubated for 4–6 days at room temperature in darkness. Fusarium-like colonies were transferred to new PDA plates. The selection of Fusarium-like colonies was based on morphological characteristics and the color of aerial mycelium.

For storage purposes, colonies were transferred to new PDA plates, cultured for 6 days and covered with 1.5 g of sterile soil. Fungi were cultured at room temperature for 7–14 days until mycelium had overgrown the soil. A total of 43 *Fusarium* isolates were assigned with unique isolate codes and are stored at –25 °C in the fungal collection of the Department of Botany and Nature Protection, University of Warmia and Mazury in Olsztyn, Poland.

### 3.2. DNA Isolation and Species Identification

A patch of mycelium (approximately 0.1–0.2 mg) was scraped from the PDA plate and transferred to homogenization tubes with 1 mm silica spheres (Lysing matrix C, MP Biomedicals, Santa Ana, CA, USA). DNA extraction was performed using a ChargeSwitch® gDNA Plant Kit (Invitrogen, Carlsbad, CA, USA). Homogenization was conducted using a FastPrep-24 instrument (MP Biomedicals, Santa Ana, CA, USA).

The FungiQuant assay [21] was used to check the total extracted DNA. Positive signals of amplification in all analyzed samples indicated that all extracted DNA can be examined with different real-time PCR assays (Table 2) to assign fungal species and mycotoxin genotypes. 

Enniatin genotypes were determined using TaqMan assay targeting the esyn1 gene [26]. Trichothecene genotype of single *F. graminearum* s.s. strain was determined using TaqMan assays targeting the Tri12 gene [27]. All reactions were performed in three replicates.

## Figures and Tables

**Table 1:** Documented incidence of grain-associated Fusaria on common vetch, faba bean, and blue and white lupine.

*Fusarium* Species	Host	Geographic Location	Sampling Season	References
*F. acuminatum*	Common vetch	Canada	-	[7]
*F. acutatum*	Faba bean	Sudan	-	[8]
*F. avenaceum*	Blue lupine	Poland	2011–2013	[9]
	Common vetch	-	-	[10]
	Faba bean	Ethiopia	1993	[11]
	Faba bean	Poland	1981–1984	[12]
	Faba bean	Poland	2001	[13]
	Faba bean	Poland	2010–2011	[14]
	White lupine	Poland	2011	[9]
*F. culmorum*	Faba bean	Poland	1981–1984	[12]
	Faba bean	Poland	2001	[13]
	Faba bean	Poland	2010–2011	[14]
*F. compactum*	Faba bean	Sudan	-	[8]
*F. equiseti*	Blue lupine	Poland	2012	[9]
	Faba bean	Poland	1981–1984	[12]
	Faba bean	Poland	2010–2011	[14]
	White lupine	Poland	2011	[9]
*F. graminearum*	Faba bean	Poland	2001	[13]
*F. nygamai*	Faba bean	Sudan	-	[8]
*F. oxysporum*	Blue lupine	Poland	2010–2012	[15]
	Common vetch	Canada	-	[7]
	Faba bean	Poland	1981–1984	[12]
	Faba bean	United Kingdom	1973	[16]
	Faba bean	Sudan	-	[8]
	Faba bean	Poland	2001	[13]
	Faba bean	Egypt	2004–2005	[17]
	Faba bean	Ethiopia	2010–2011	[18]
	Faba bean	Poland	2010–2011	[14]
	Faba bean	Egypt	-	[19]
*F. poae*	Blue lupine	Poland	2012–2013	[9]
	White lupine	Poland	2011–2012	[9]
*F. proliferatum*	Faba bean	Sudan	-	[8]
	White lupine	Croatia	-	[20]
*F. semitectum*	Faba bean	Sudan	-	[8]
	Faba bean	Egypt	2004–2005	[17]
*F. solani*	Faba bean	Poland	1981–1984	[12]
	Faba bean	United Kingdom	1973	[16]
	Faba bean	Sudan	-	[8]
	Faba bean	Egypt	2004–2005	[17]
	Faba bean	Poland	2010–2011	[14]
	Faba bean	Ethiopia	2010–2011	[18]
*F. sporotrichioides*	Blue lupine	Poland	2013	[9]
	Faba bean	Poland	2001	[13]
	Faba bean	Poland	2010–2011	[14]
*F. tricinctum*	Blue lupine	Poland	2012–2013	[9]
*F. verticillioides*	Common vetch	Croatia	-	[20]
	Faba bean	Egypt	2004–2005	[17]
	White lupine	Croatia	-	[20]

(-)—data not available.

**Table 2:** List of real-time polymerase chain reaction (PCR) assays used to determine species, trichothecene genotypes, and enniatin genotypes.

Specificity of the qPCR Assay	Primer/Probe Sequence	Reaction Reagents	Reaction Conditions	References
Total fungal DNA				
FungiQuant	F: GGRAAACTCACCAGGTCCAG	A	95 °C for 20 s, (95 °C for 1 s, 60 °C for 30 s) × 40	[21]
	R: GSWCTATCCCCAKCACGA			
	Probe: (6FAM)-TGGTGCATGGCCGTT-(MGBNFQ)			
Species				
*F. avenaceum*	F: CCATCGCCGTGGCTTTC R: CAAGCCCACAGACACGTTGT Probe: FAM-ACGCAATTGACTATTGC-MGB	B	95 °C for 20 s, (95 °C for 1 s, 60 °C for 50 s) × 40	[22]
*F. culmorum*	F: TCGTTGACGGTGAGGGTTGT R:GACTCGAACACGTCAACCAACT Probe: FAM-CGGTTATTATTTCGAAAAGT- MGB	A	95 °C for 20 s, (95 °C for 1 s, 60 °C for 30 s) × 40	[23]
*F. equiseti*	F: CACCGTCATTGGTATGTTGTCATC R: TGTTAGCATGAGAAGGTCATGAGTG	C	95 °C for 5 min, (95 °C for 15 s, 65 °C for 60 s) × 40, dissociation curve analysis at 60–95 °C.	[24]
*F. graminearum* s.s.	F: TGGCCTGAATGAAGGATTTCTAG R: CATCGTTGTTAACTTATTGGAGATG Probe: FAM-TTAAACACTCAAACACTACA- MGB	A	95 °C for 20 s, (95 °C for 1 s, 60 °C for 30 s) × 40	[25]
*F. langsethiae*	F: CAAGTCGACCACTGTGAGTACCTCT R: TGTCAAAGCATGTCAGTAAAGATGAC	C	95 °C for 5 min, (95 °C for 15 s, 65 °C for 60 s) × 40, dissociation curve analysis at 60–95 °C.	[24]
*F. poae*	F: AAATCGGCGTATAGGGTTGAGATA R: GCTCACACAGAGTAACCGAAACCT Probe: FAM-CAAAATCACCCAACCGACCCTTTC-TAMRA	B	50 °C for 2 min, 95 °C for 10 min, (95 °C for 15 s, 60 °C for 60 s) × 40	[22]
*F. proliferatum*	F: CTTCGATCGCGCGTCCT R: CACGTTTCGAATCGCAAGTG	C	95 °C for 5 min, (95 °C for 15 s, 65 °C for 60 s) × 40, dissociation curve analysis at 60–95 °C.	[24]
*F. sporotrichioides*	F: GCAAGTCGACCACTGTGAGTACA R: CTGTCAAAGCATGTCAGTAAAAATGAT	C	95 °C for 5 min, (95 °C for 15 s, 65 °C for 60 s) × 40, dissociation curve analysis at 60–95 °C.	[24]
*F. subglutinans*	F: TCATTGGTATGTTGTCGCTCATG R: GTGATATGTTAGTACGAATAAAGGGAGAAC	C	95 °C for 5 min, (95 °C for 15 s, 65 °C for 60 s) × 40, dissociation curve analysis at 60–95 °C.	[24]
F. *verticillioides*	F: CGTTTCTGCCCTCTCCCA R: TGCTTGACACGTGACGATGA	C	95 °C for 5 min, (95 °C for 15 s, 65 °C for 60 s) × 40, dissociation curve analysis at 60–95 °C.	[24]
Enniatin genotype				
*esyn1*	F: AGCAGTCGAGTTCGTCAACAGA R: GGCYTTTCCTGCGAACTTG Probe: FAM-CCGTCGAGTCCTCT-MGB	B	95 °C for 20 s, (95 °C for 3 s, 60 °C for 30 s) × 40	[26]
*Tri* genotypes				
3ADON	F: CATGCGGGACTTTGATCGAT	B	95 °C for 20 s, (95 °C for 1 s, 60 °C for 50 s) × 40	[27]
	R: TTTGTCCGCTTTCTTTCTATCATAAA			
	Probe: FAM-CTCACCGATCATGTTC-MGB			
15ADON	F: TCCAATCATTGCCAGCCTCTA			
	R: TGATGCGGAACATGGTCTGT			
	Probe: FAM-ATGAGGGACTTTGACCAAT-MGB			
NIV	F: TCGCCAGTCTCTGCATGAAG			
	R: CCTTATCCGCTTTCTTTCTATCATAAA			
	Probe: FAM-CTGATCATGTCCCGCATC-MGB			

A 2 µL gDNA, 14.3 µL H2O, 6.7 µM of each primer, 1.7 µM of probe, 3.6 µL TaqMan Fast Advanced Master Mix (Applied Biosystems, Foster City, CA, USA). B 2 µL gDNA, 10.8 µL H2O, 6.7 µM of each primer, 1.7 µM of probe, 7.2 µL TaqMan Fast Advanced Master Mix (Applied Biosystems, Foster City, CA, USA). C 2 µL gDNA, 8.5 µL H2O, 1 µM of each primer, 12.5 µL 2× SYBR Green PCR Master Mix (Applied Biosystems, Foster City, CA, USA).

**Table 3:** List of identified Fusarium species in different legume grains in Poland.

Plant Host	*F. avenaceum*	*F. equiseti*	*F. graminearum s.s.*	*F. sporotrichioides*	*F. tricinctum*	Not Identified
Blue lupine	7	-	-	3	-	1
Common vetch	7	9	1	-	1	-
Faba bean	7	-	-	1	-	4
White lupine	-	1	-	-	-	1

(-)—no positive results.

## Supplementary Materials

The following are available online at https://www.mdpi.com/2072-6651/11/10/569/s1, Table S1: Species and mycotoxin genotype identification of *Fusarium* isolates using real-time PCR assays.
