# Supplementary material for: A Preliminary Survey of Cultured Fusaria from Symptomatic Legume Grains in North-Eastern Poland
Source: Toxins (Basel). 2019 Sep 29;11(10):569. doi: 10.3390/toxins11100569 (PMC6832508; doi:10.3390/toxins11100569)
Supplement: Supplementary file 1 [file toxins-11-00569-s001.pdf]

Supplementary Materials: A Preliminary Survey of Cultured Fusaria from Symptomatic Legume Grains in North-Eastern Poland

Maciej Żelechowski, Jacek Olszewski and Tomasz Kulik

Table S1. Species and mycotoxin genotype identification of *Fusarium* isolates using real-time PCR assays.

Fungal Species	Strain code	Geographical Origin	Host Plant (Species/Variety)	Year of Isolation	Fungi Quant [21]	*F. avenaceum* [22]	*F. culmorum* [23]	*F. equiseti* [24]	*F. graminearum s.s.* [25]	*F. langsethiae* [24]	*F. poae* [22]	*F. proliferatum* [24]	*F. sporotrichioides* [24]	*F. subglutinans* [24]	*F. verticillioides* [24]	Enniatin Genotype [26]	Trichothecene Genotypes [27]
					CT (cycle threshold) Mean ± CT SD (standard deviation)												
F. avenaceum	W18/6	Wrócikowo	Common Vetch/Greta	2018	10.6 ± 0.17	19.9 ± 0.09	n.t.	n.t.	n.t.	n.t.	n.t.	n.t.	n.t.	n.t.	n.t.	16.2 ± 1.49	n.t.
	W18/7	Wrócikowo	Common Vetch/Greta	2018	11 ± 0.16	20.4 ± 0.49	n.t.	n.t.	n.t.	n.t.	n.t.	n.t.	n.t.	n.t.	n.t.	16.2 ± 0.28	n.t.
	W18/13	Wrócikowo	Common Vetch/Ina	2018	12 ± 0.41	21 ± 0.12	n.t.	n.t.	n.t.	n.t.	n.t.	n.t.	n.t.	n.t.	n.t.	15.8 ± 0.04	n.t.
	W18/14	Wrócikowo	Common Vetch/Ina	2018	12.9 ± 0.09	21.6 ± 0.10	n.t.	n.t.	n.t.	n.t.	n.t.	n.t.	n.t.	n.t.	n.t.	20.1 ± 0.11	n.t.
	W18/15	Wrócikowo	Common Vetch/Greta	2018	10.1 ± 0.11	19.5 ± 0.10	n.t.	n.t.	n.t.	n.t.	n.t.	n.t.	n.t.	n.t.	n.t.	18.3 ± 0.27	n.t.
	W18/17	Wrócikowo	Common Vetch/Greta	2018	11.3 ± 0.21	19.30 ± 0.27	n.t.	n.t.	n.t.	n.t.	n.t.	n.t.	n.t.	n.t.	n.t.	16.2 ± 0.46	n.t.
	W18/18	Wrócikowo	Common Vetch/Ina	2018	11.5 ± 0.07	19.10 ± 0.46	n.t.	n.t.	n.t.	n.t.	n.t.	n.t.	n.t.	n.t.	n.t.	19.6 ± 0.36	n.t.
	Ł18/1	Biskupiec	Blue lupin/Regent	2018	13.3 ± 0.17	20.8 ± 0.29	n.t.	n.t.	n.t.	n.t.	n.t.	n.t.	n.t.	n.t.	n.t.	16.3 ± 0.36	n.t.
	Ł18/2	Biskupiec	Blue lupin/Regent	2018	11.9 ± 0.14	20.4 ± 0.43	n.t.	n.t.	n.t.	n.t.	n.t.	n.t.	n.t.	n.t.	n.t.	16.2 ± 0.11	n.t.
	Ł18/3	Biskupiec	Blue lupin/Regent	2018	14.7 ± 0.35	21.3 ± 0.64	n.t.	n.t.	n.t.	n.t.	n.t.	n.t.	n.t.	n.t.	n.t.	17.78 ± 0.22	n.t.
	Ł18/4	Biskupiec	Blue lupin/Regent	2018	13.7 ± 0.22	19.4 ± 0.33	n.t.	n.t.	n.t.	n.t.	n.t.	n.t.	n.t.	n.t.	n.t.	16 ± 0.19	n.t.
	Ł18/5	Biskupiec	Blue lupin/Regent	2018	13.5 ± 0.44	20 ± 0.09	n.t.	n.t.	n.t.	n.t.	n.t.	n.t.	n.t.	n.t.	n.t.	16.6 ± 0.16	n.t.
	Ł18/6	Biskupiec	Blue lupin/Regent	2018	14.5 ± 0.07	20.9 ± 0.01	n.t.	n.t.	n.t.	n.t.	n.t.	n.t.	n.t.	n.t.	n.t.	18.6 ± 1.08	n.t.
	Ł18/7	Biskupiec	Blue lupin/Regent	2018	14.6 ± 0.12	21.1 ± 0.06	n.t.	n.t.	n.t.	n.t.	n.t.	n.t.	n.t.	n.t.	n.t.	17.8 ± 0.10	n.t.

	B18/1	Barciany	Faba bean/Bobas	2018	10.4 ± 0.17	20.2 ± 0.25	n.t.	n.t.	n.t.	n.t.	n.t.	n.t.	n.t.	n.t.	n.t.	15.9 ± 0.27	n.t.
	B18/2	Barciany	Faba bean/Bobas	2018	12 ± 0.09	20.5 ± 0.19	n.t.	n.t.	n.t.	n.t.	n.t.	n.t.	n.t.	n.t.	n.t.	15.7 ± 0.10	n.t.
	B17/2	Lidzbark Warmiński	Faba bean/Nadwiślański	2017	11.8 ± 0.37	20.4 ± 0.13	n.t.	n.t.	n.t.	n.t.	n.t.	n.t.	n.t.	n.t.	n.t.	16.3 ± 0.23	n.t.
	B17/15	Barciany	Faba bean/Nadwiślański	2017	13.2 ± 0.15	21.3 ± 0.21	n.t.	n.t.	n.t.	n.t.	n.t.	n.t.	n.t.	n.t.	n.t.	17.4 ± 0.33	n.t.
	B17/18	Barciany	Faba bean/Nadwiślański	2017	15.5 ± 0.08	23.6 ± 0.17	n.t.	n.t.	n.t.	n.t.	n.t.	n.t.	n.t.	n.t.	n.t.	19.2 ± 0.21	n.t.
	B17/22	Barciany	Faba bean/Nadwiślański	2017	15.8 ± 1.21	24.6 ± 2.64	n.t.	n.t.	n.t.	n.t.	n.t.	n.t.	n.t.	n.t.	n.t.	17.1 ± 0.06	n.t.
	B17/27	Sępopol	Faba bean/Nadwiślański	2017	10.9 ± 0.24	19.8 ± 0.02	n.t.	n.t.	n.t.	n.t.	n.t.	n.t.	n.t.	n.t.	n.t.	16.4 ± 0.91	n.t.
	W18/1	Wrócikowo	Common Vetch/Ina	2018	11.9 ± 0.20	-	-	16.05 ± 0.10	-	n.t.	-	n.t.	n.t.	n.t.	n.t.	n.t.	n.t.
	W18/3	Wrócikowo	Common Vetch/Ina	2018	13.8 ± 0.17	-	-	16.15 ± 0.07	-	n.t.	-	n.t.	n.t.	n.t.	n.t.	n.t.	n.t.
	W18/4	Wrócikowo	Common Vetch/Ina	2018	12.2 ± 0.02	-	-	16.48 ± 0.13	-	n.t.	-	n.t.	n.t.	n.t.	n.t.	n.t.	n.t.
	W18/8	Wrócikowo	Common Vetch/Ina	2018	12.6 ± 0.14	-	-	17.34 ± 0.03	-	n.t.	-	n.t.	n.t.	n.t.	n.t.	n.t.	n.t.
F. equiseti	W18/9	Wrócikowo	Common Vetch/Ina	2018	12.3 ± 0.15	-	-	16.49 ± 0.05	-	n.t.	-	n.t.	n.t.	n.t.	n.t.	n.t.	n.t.
	W18/10	Wrócikowo	Common Vetch/Ina	2018	12.8 ± 0.06	-	-	16.93 ± 0.17	-	n.t.	-	n.t.	n.t.	n.t.	n.t.	n.t.	n.t.
	W18/11	Wrócikowo	Common Vetch/Ina	2018	12.5 ± 0.49	-	-	16.61 ± 0.21	-	n.t.	-	n.t.	n.t.	n.t.	n.t.	n.t.	n.t.
	W18/12	Wrócikowo	Common Vetch/Ina	2018	11.9 ± 0.21	-	-	16.35 ± 0.17	-	n.t.	-	n.t.	n.t.	n.t.	n.t.	n.t.	n.t.
	W18/16	Wrócikowo	Common Vetch/Greta	2018	11.4 ± 0.15	-	-	15.87 ± 0.31	-	n.t.	-	n.t.	n.t.	n.t.	n.t.	n.t.	n.t.
	Ł17/1	Tomaszkowo	White lupin/Butan	2017	13 ± 0.22	-	-	16.40 ± 0.11	-	n.t.	-	n.t.	n.t.	n.t.	n.t.	n.t.	n.t.
F. graminearum s.s.	W18/5	Wrócikowo	Common Vetch/Ina	2018	12.5 ± 0.14	-	-	n.t.	18.8 ± 0.15	n.t.	n.t.	n.t.	n.t.	n.t.	n.t.	n.t.	3ADON (17.5 ± 0.15) 15ADO

																	N (-) NIV (-)
F. sporotrichioides	Ł18/8	Ełk district	Blue lupin/Zeus	2018	11.7 ± 0.15	-	-	-	-	n.t.	-	n.t.	20.71 ± 0.27	n.t.	n.t.	n.t.	n.t.
	Ł18/10	Ełk district	Blue lupin/Zeus	2018	14.1 ± 0.13	-	-	-	-	n.t.	-	n.t.	22.47 ± 2.73	n.t.	n.t.	n.t.	n.t.
	Ł18/11	Ełk district	Blue lupin/Zeus	2018	12.1 ± 0.25	-	-	-	-	n.t.	-	n.t.	21.06 ± 0.85	n.t.	n.t.	n.t.	n.t.
	B17/8	Barciany	Faba bean/Nadwiślański	2017	11.3 ± 0.06	-	-	-	-	n.t.	-	n.t.	20.78 ± 0.48	n.t.	n.t.	n.t.	n.t.
F. tricinctum	W18/2	Wrócikowo	Common Vetch/Ina	2018	13 ± 0.08	-	-	-	-	-	-	-	-	-	-	28.4 ± 0.04	n.t.
**Not identified**	B17/28	Sępopol	Faba bean/Nadwiślański	2017	11.6 ± 0.06	-	-	-	-	-	-	-	-	-	-	-	n.t.
	B17/32	Tomaszkowo	Faba bean/Albus	2017	17.2 ± 0.19	-	-	-	-	-	-	-	-	-	-	-	n.t.
	B17/33	Tomaszkowo	Faba bean/Albus	2017	12.8 ± 0.08	-	-	-	-	-	-	-	-	-	-	-	n.t.
	B17/34	Tomaszkowo	Faba bean/Albus	2017	12 ± 0.19	-	-	-	-	-	-	-	-	-	-	-	n.t.
	Ł18/9	Ełk district	Blue lupin/Zeus	2018	12.6 ± 0.08	-	-	-	-	-	-	-	-	-	-	-	n.t.
	Ł17/2	Tomaszkowo	White lupin/Butan	2017	15.8 ± 0.17	-	-	-	-	-	-	-	-	-	-	-	n.t.

(-)—negative result. n.t.—not tested; []—numbers in square brackets correspond to references in main manuscript.
